# Effect of antimicrobial peptides on planktonic growth, biofilm formation and biofilm-derived bacterial viability of *Streptococcus pneumoniae*

**DOI:** 10.4102/sajid.v36i1.226

**Published:** 2021-01-25

**Authors:** Michael T. Boswell, Riana Cockeran

**Affiliations:** 1 Department of Internal Medicine, Division of Infectious Diseases, Faculty of Medicine, Steve Biko Academic Hospital, Pretoria, South Africa; 2 Department of Immunology, Faculty of Health Sciences, University of Pretoria, Pretoria, South Africa; 3 Department of Immunology, Tshwane Academic Division, National Health Laboratory Services, Pretoria, South Africa

**Keywords:** antimicrobial peptides, *Streptococcus pneumoniae*, LL37, biofilm, cathelicidins, bacterial growth

## Abstract

*Streptococcus pneumoniae* is a leading cause of pneumonia mortality globally. Pneumococcal disease is often associated with prolonged colonisation of hosts and this process is facilitated by biofilm formation that is largely resistant to conventional antibiotics. We investigated the effects of antimicrobial peptides (AMPs) lysozyme, lactoferrin, LL37 and a combination of all three on planktonic growth, biofilm formation and biofilm-derived bacterial viability by *S. pneumoniae*, serotype 23F. Planktonic growth and biofilm-derived bacterial viability were determined using standard colony-forming techniques, while biofilm formation was measured using a crystal violet based spectrophotometric method. Relative to controls, lysozyme significantly reduced biofilm formation (0.08 OD vs. 0.10 OD at 570 nm, *p* = 0.01), while LL37 and the AMP combination increased biofilm formation (0.14 OD vs. 0.10 OD at 570 nm, *p* = 0.01). The combination of AMPs significantly decreased planktonic growth (1.10 × 10^8^ colony-forming units per millilitres [CFU/mL] vs. 2.13 × 10^8^ CFU/mL, *p* = 0.02). Biofilm-derived bacterial viability was greatly reduced by exposure to a combination of AMPs (1.05 × 10^5^ CFU/mL vs. 1.12 × 10^6^ CFU/mL, *p* = 3.60 × 10^−8^). *Streptococcus pneumoniae* displays marked resistance to the individual AMPs. A combination of lysozyme, lactoferrin and LL37 effectively inhibited planktonic growth and biofilm-derived bacterial viability; however, persister cell growth was still evident after exposure.

## Introduction

*Streptococcus pneumoniae* is an immense public health problem. In preceding decades, expanded vaccination and antimicrobial therapy have led to an impressive reduction in pneumococcal-associated morbidity and mortality. Despite this, *S. pneumoniae* is the leading cause of mortality for lower respiratory tract infections globally.^1^ *Streptococcus pneumoniae* colonises hosts by forming biofilms in the respiratory tract, and the biofilm-derived bacteria are resistant to antibiotic therapy.^2^ A biofilm refers to a mushroom-shaped colony of bacteria adherent to a surface and encased in an extracellular matrix composed of a variety of polymers including, but not limited to, adhesion molecules, pili, protein binding carbohydrates and extracellular deoxyribonucleic acid (DNA) derived from dead bacteria.^3^ A biofilm’s development and dispersal are regulated by quorum-sensing mechanisms that control bacterial colonies’ growth, gene expression and metabolism in response to environmental and internal stimuli.^4^ Biofilms are therefore an important contributor to bacterial survival following exposure to antibiotics and can promote the emergence of antibiotic resistance through persister cells that survive the initial exposure.^5^

Antimicrobial peptides (AMPs) are produced by cells involved in innate immunity and are present at mucosal surfaces. They have become popular topics in pharmaceutical research because of their diverse antimicrobial mechanisms of action, relatively low toxicity to human cells and potential for synergism with conventional antibiotics.^6^ Antimicrobial peptides are cationic and amphipathic molecules which vary widely with respect to structure and function. The three main classes of AMPs important to the mammalian immune system are defensins, cathelicidins and histidins.^7^ LL37 is a 37 amino acid long peptide cathelicidin which forms pores in bacterial cell walls. Lysozyme and lactoferrin are larger molecules found in respiratory tract secretions. The antibacterial mechanism of action of these molecules varies substantially, and includes degrading bacterial cell walls (muramidase activity common to lysozyme and LL37), sequestering iron required for normal bacterial metabolism (lactoferrin), interfering with bacterial cell attachment and quorum-sensing mechanisms (LL37).^8,9,10^ These mechanisms make antimicrobial peptides (AMPs) attractive options as antibiofilm agents, and to our knowledge their activity against pneumococcal biofilms has not been investigated. Furthermore, it is unclear whether they can penetrate biofilms and kill persister cell bacteria.

The purpose of this study was to investigate the effect of AMPs derived from the human respiratory tract on planktonic growth (i.e. free-living bacteria in a liquid medium), biofilm formation and biofilm-derived bacterial viability of *S. pneumoniae*.

## Materials and methods

Unless otherwise specified, all chemicals and reagents were purchased from Sigma Chemical Co. (St Louis, MO, USA).

### Antimicrobial peptides

The following AMPs were used at physiologically relevant concentrations: lysozyme (100 micrograms per millilitres [µg/mL]), lactoferrin (70 µg/mL) and LL37 (20 µg/mL). In addition, a combination of all three peptides was also tested. Appropriate solvent controls (sterile phosphate buffered saline [PBS]) were included in all experiments.

### Bacterial strain

An *S. pneumoniae* macrolide sensitive, South African clinical isolate, strain 172, serotype 23F, multilocus sequence type 81 was provided by the National Institute for Communicable Diseases (NICD) and used for the purpose of this study. Bacterial seed cultures were stored at −70°C and used as required.

### Planktonic growth

Bacterial seed cultures were used as inoculums and grown to a mid-logarithmic phase in Tryptone Soy broth (TSB, Merck, Dramstadt, Germany), the inoculums were centrifuged for 15 min at 1912 g and the concentrated bacterial pellet resuspended in TSB before optical standardisation (Powerwave X, Bio-Tec Instruments Inc., Winooski, VT, USA) equivalent to 1.8 × 10^6^ colony-forming units (CFU)/mL. The standardised bacterial cultures were then exposed to AMPs, individually or in combination, for 16 h at 37°C, 5% CO_2_ in a humidified incubator (Hotpack Incubator, Hotpack Corporation, Philadelphia, PA, USA). Following incubation, growth was analysed using standardised CFU procedures. To measure CFU the bacterial cultures were serially diluted sixfold in PBS; these dilutions were then used to inoculate blood agar plates. Individual colonies were manually counted, and the number was converted to CFU/mL.

### Biofilm formation

The pneumococci, treated with or without AMPs, were placed in six-well tissue culture plates for 16 h at 37°C, 5% CO_2_ (Hotpack), to facilitate biofilm formation. Following incubation, the unbound bacteria were removed, and the biofilm was washed with PBS. The total biofilm was stained with 0.1% crystal violet for 20 min, followed by the release of the dye (using 96% ethanol) and detection (absorbance determination at 570 nanometres [nm] – Powerwave X) of the biofilm-associated stain. The amount of bound crystal violet correlated with the amount of biofilm formed.^11^

### Biofilm-derived bacterial viability

The pneumococci, in the absence of AMPs, were placed in six-well tissue culture plates in the presence of glass beads for 16 h at 37°C, 5% CO_2_ (Hotpack) to facilitate biofilm formation. The unbound bacteria were removed and the biofilm-encased bacteria exposed to PBS or the AMPs in PBS for a period of 6 h at 37°C, with 5% CO_2_. The biofilm was then disrupted through gentle sonification (UMC2, Integral Systems, Randburg, South Africa) for 10 min. Viability of bacteria within the biofilm was defined as the number of live bacteria determined through standardised CFU procedures, as described above, derived from the disrupted biofilm.

### Statistical analysis

This study assessed five treatment conditions with respect to their effect on pneumococcal growth, biofilm formation and biofilm-derived bacterial viability. Each experimental procedure was repeated three times with three to six replicates per treatment modality. The data were non-parametric and therefore summarised with the median and interquartile range (IQR). Results from the treated systems were compared to the untreated controls in R studio^12,13^ using an unpaired Mann–Whitney *U*-test (MW). Results were visualised using pirate plots in R.^14^ Statistical significance was determined as *p* < 0.05, corrected for multiple comparison using the Holm–Bonferroni method.

### Ethical consideration

This article followed all ethical standards for carrying out research, and was approved by the University of Pretoria research ethics committee.

## Results

### Effects of the antimicrobial peptides on the growth of planktonic *S. pneumoniae*

**FIGURE 1:**
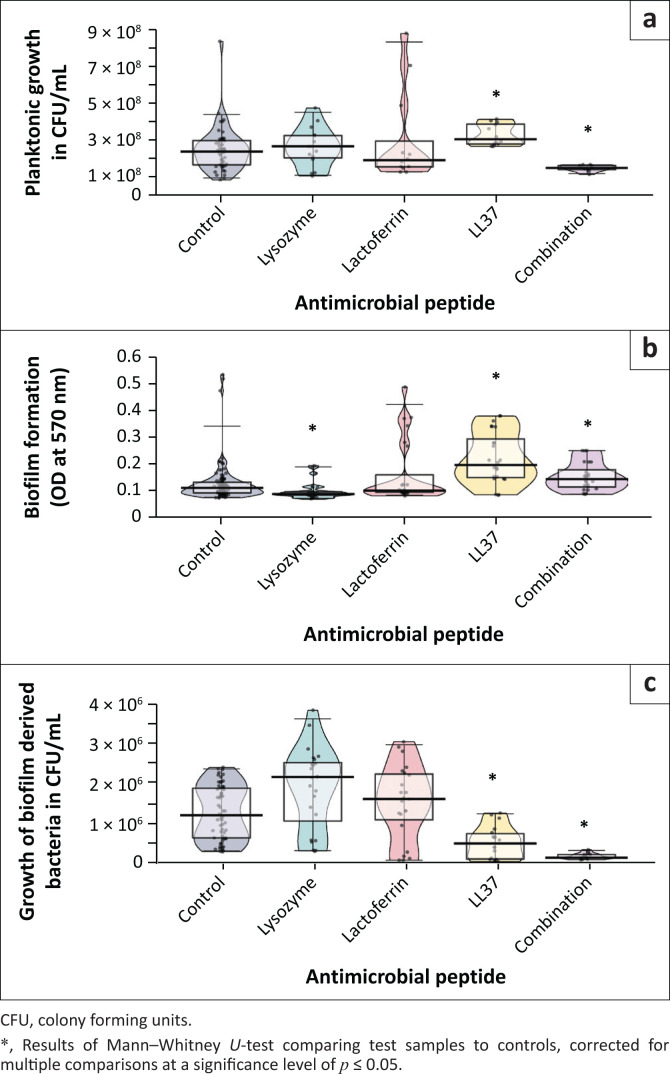
The effects of antimicrobial peptides alone and in combination on planktonic growth (a), biofilm formation (b), and biofilm-derived bacterial viability (c) of *S. pneumoniae* strain 172. Lysozyme (100 micrograms per millilitres [µg/mL]), lactoferrin (70 µg/mL) and LL37 (20 µg/mL). The results of three to four experiments with three to six replicates per system are presented as the pirate plots, with the median and interquartile range shown.

The effects of the AMPs, alone and in combination, on the growth (expressed as CFU/mL) of *S. pneumoniae* are shown in Figure 1a. Exposure to LL37 was associated with increased planktonic growth relative to controls (2.90 × 10^8^ CFU/mL vs. 2.13 × 10^8^ CFU/mL, MW *p* = 0.02). A combination of AMPs significantly reduced planktonic bacterial growth (1.10 × 10^8^ CFU/mL vs. 2.13 × 10^8^ CFU/mL, MW *p* = 0.02).

### Effects of the antimicrobial peptides on biofilm formation of *S. pneumoniae*

The amount of total biofilm formed (expressed as an optical density [OD] at 570 nm – after the subtraction of the background) in the presence of the selected AMPs individually, as well as in combination, is shown in Figure 1b. Lysozyme significantly reduced the amount of biofilm formed (0.08 OD vs. 0.10 OD at 570 nm, MW *p* = 0.01) and LL37 greatly increased biofilm formation (0.19 OD vs. 0.10 OD at 570 nm, MW *p* = 9.60 × 10^−6^). Exposure to the combination of AMPs enhanced biofilm formation significantly, though to a lesser degree when compared to LL37 on its own (0.14 OD vs. 0.10 OD at 570 nm, MW *p* = 0.01).

### Effects of antimicrobial peptides on the biofilm-derived bacterial viability

The effects of AMPs on the viability of bacteria within the preformed biofilm are shown in Figure 1c. Exposure to lysozyme was associated with increased growth of biofilm-derived bacteria, though this was not statistically significant after correction for multiple comparison (2.14 × 10^6^ vs. 1.12 × 10^6^, MW *p* = 0.07). LL37 alone (4.60 × 10^5^ CFU/mL vs. 1.12 × 10^6^ CFU/mL, MW *p* = 8.60 × 10^−4^) and in combination with lysozyme and lactoferrin decreased biofilm viability, and these results were highly significant (1.05 × 10^5^ CFU/mL vs. 1.12 × 10^6^ CFU/mL, MW *p* = 3.60 × 10^−8^). Importantly, a persistent population of bacteria survived exposure to the AMP combination.

## Discussion

Growth of planktonic *S. pneumoniae* was significantly increased by exposure to LL37; however, the combination of lysozyme, lactoferrin and LL37 decreased planktonic growth significantly. *Streptococcus pneumoniae* has developed several resistance mechanisms against AMPs, and therefore a combination of AMPs appears to be necessary for effective antimicrobial action.^15^ Lactoferrin exposure has been associated with enhanced growth of *S. pneumoniae* serotypes 3 and 6B; however, the concentrations used in these experiments were tenfold higher compared to those presented here. The authors speculated that lactoferrin may be used as an iron source for bacterial metabolism and thereby enhance growth.^16^

Cell wall components are an important part of biofilm structure. Lysozyme may decrease biofilm formation by degrading these compounds via its muramidase action.^17^ Biofilm formation was significantly increased by LL37 as well as the combination of lysozyme, lactoferrin and LL37. The increased biofilm formation after LL37 exposure may be caused by increased bacterial growth. Alternatively, the increase in biofilm formation may be related to bacterial shedding of the autolysin LytA. LytA is localised to the bacterial cell wall, and in response to LL37 exposure LytA will activate and *S. pneumoniae* will shed its capsule – the constituents of which are then able to provide a scaffold for biofilm formation.^18^ Therefore, the increase in biofilm formation by the combination of lysozyme, lactoferrin and LL37 may result from AMPs acting as a stressor. Exposure to the combination of AMPs might change gene expression profiles to favour biofilm formation – but this requires further investigation. In addition, this may be an evolved mechanism of *S. pneumoniae* to enhance its colonisation ability, as these AMPs are constitutively expressed in the human respiratory system.^19^

Exposure of a preformed biofilm to lysozyme may increase the growth of *S. pneumoniae* within a biofilm. This finding may appear counterintuitive but is not without precedence. *Staphylococcus aureus* when exposed to lysozyme exhibits a prolonged logarithmic growth phase, and may enhance growth as a result.^20^ However, these findings may not be relevant to *S. pneumoniae*. An alternative explanation for these findings is that *S. pneumoniae* in biofilm metabolises amino acids preferentially over carbohydrates as an energy source. This could promote bacterial growth in biofilms if resistance to the AMP is present, as is the case with lysozyme.^21^ Lysozyme and LL37 have a synergistic antimicrobial action, and lysozyme and lactoferrin have an additive effect.^22^ The mechanism underlying the synergistic effects of the above AMPs may be because of increased stress placed on the bacterial cell wall – this is one of the known shared antimicrobial effects of LL37 and lysozyme.^23^ This may explain why the combination of AMPs decreased the viability of *S. pneumoniae* so notably.

Our experiments have several limitations, these include: (1) a limited range of AMP concentrations were used because of cost constraints. (2) Only a single serotype and strain was used; significant differences exist in the susceptibility of the different pneumococcal serotypes and strains to AMPs. Therefore, these results will not necessarily be relevant to other strains or serotypes. (3) It has been shown that *S. pneumoniae* grows a biofilm that is more stable and resistant to antibiotics when using *in vivo* models, or using human epithelial cell cultures as opposed to *in vitro* models. These experiments may not correlate with *in vivo* results.^24^ Further limitations of this study include the fact that the AMPs were tested against biofilm in a PBS deprived of nutrients. This was done to decrease the replication of planktonic bacteria from the formed biofilms, which may have made interpretation of the effects of the AMPs on the biofilm difficult. In addition, while we can comment on the viability of bacteria within the biofilm, we did not evaluate the effect of the selected AMPs on the structure of the biofilm, which may have important implications for further research.

## Conclusion

In summary, we show that *S. pneumoniae* is largely resistant to individual AMPs and may be able to use them as a source of nutrients. However, a combination of lysozyme, lactoferrin and LL37 not only reduced planktonic growth but was also able to penetrate a biofilm and decrease the viability of bacteria within it. However, persister cells survived exposure to a combination of AMPs. The tested AMPs have a significant effect on multiple aspects of *S. pneumoniae* biology. These AMPs may be important in determining whether *S. pneumoniae* successfully colonises a host, and whether a colonised host develops invasive pneumococcal-associated disease.
